# A renal aplasia case mimicking radiologically as unilateral renal agenesis in a child with spina bifida, atresia ani and unilateral undescended testis: a case report

**DOI:** 10.1186/s13256-023-04330-0

**Published:** 2024-01-26

**Authors:** Prahara Yuri, Muhammad Anwar Irzan, Tanaya Ghinorawa, Muchamad Ridotu Solichin, Ery Kus Dwianingsih

**Affiliations:** 1 Division of Urology, Department of Surgery, Faculty of Medicine, Public Health and Nursing, Universitas Gadjah Mada/Dr. Sardjito Hospital, Jl. Kesehatan No.1, Yogyakarta, 55281 Indonesia; 2 Department of Anatomical Pathology, Faculty of Medicine, Public Health and Nursing UGM/Dr. Sardjito General Hospital, Yogyakarta, Indonesia

**Keywords:** Unilateral renal agenesis, Severe kidney dysplasia, Aplastic kidney, Immunohistochemistry

## Abstract

**Background:**

As a result of the failure of embryogenic kidney formation, a condition can occur where not a single kidney appears and this phenomenon is known as unilateral renal agenesis (URA). Both aplastic and dysplastic kidney are different from renal agenesis, atrophy and renal hypoplasia. However, from this case report it can be seen that there are similarities, both radiologically and macroscopically, between cases of unilateral renal aplasia and renal agenesis.

**Case presentation:**

A 2 year old Javanese boy came to the health facility with complaints of recurrent fever and urinary tract symptoms such as dysuria and straining. Computerized Tomography (CT) scan of the abdomen and urography showed agenesis of the left kidney and a probable spina bifida. Cystourethrography examination was done and showed grade 5 voiding, then retrograde pyelography was performed with the diagnosis of unilateral renal agenesis was made because there was no visible left side collecting system even though there was a duplication in the left ureter. The next examination was carried out by histopathology and immunohistochemistry after resection of the left side of the ureter and the diagnosis increasingly pointed towards renal aplasia after primitive renal structures were found.

**Conclusions:**

Renal agenesis and aplastic kidney are difficult to differentiate macroscopically and radiologically. Nevertheless, from this case report, we try to provide some interesting points to differentiate cases of unilateral renal agenesis from Renal Dysplasia which presents as unilateral renal aplasia.

## Background

As a result of the failure of embryogenic kidney formation, a condition can occur where one kidney did not form. This phenomenon is known as unilateral renal agenesis (URA). This happens because since the embryogenic stage imperfections occur and result in the ureteral buds being unable to create the formation of ureters, renal pelvis and collecting ducts perfectly along with the renal mesenchyme being unable to form nephrons [1]. Roughly speaking, the incidence of URA occurs sporadically and the incidence is in 1 in 500–1200 birth rate [2, 3]. However, it does not rule out the possibility of URA occurring secondarily where possible causes include chromosomal abnormalities or developmental defects, such as VATER (Vertebral, Anorectal, Tracheoesophageal and Renal) abnormalities or MURCS (Mullerian ductal aplasia, uterine hypoplasia, renal aplasia anomalies, renal and cervicothoracic somitic dysplasia) [2].

Kidney dysplasia is a condition that started in the early stages of kidney development, happening during the perinatal and childhood period, where diagnosis can be made It is the abnormal differentiation of the metanephros that causes renal dysplasia [4]. The percentage of the renal dysplasia population is in the ratio of 1:1300. Sometimes, if kidney dysplasia occurs in an extreme manner, the branching can create an involution of the kidney or rudiment which is known as an aplastic kidney. Unfortunately, sometimes the appearance of a very aplastic kidney often leads to a diagnosis of solitary kidney or unilateral agenesis due to the inability of radiological examination to identify it [5].

Differentiating between renal agenesis and kidney aplasia using only macroscopic and radiological findings is difficult. The diagnosis of aplastic kidney can be confirmed when the microscopic image still shows renal parenchyma even though it is not perfect [5]. In this case report, our aim is to present a case of unilateral renal aplasia, despite the close resemblance of both radiological and macroscopic images to kidney agenesis.. The difference is an important point in the histological examination that differentiates the result of a primitive kidney picture which makes it stand out as kidney dysplasia.

## Case presentation

**Fig. 1:**
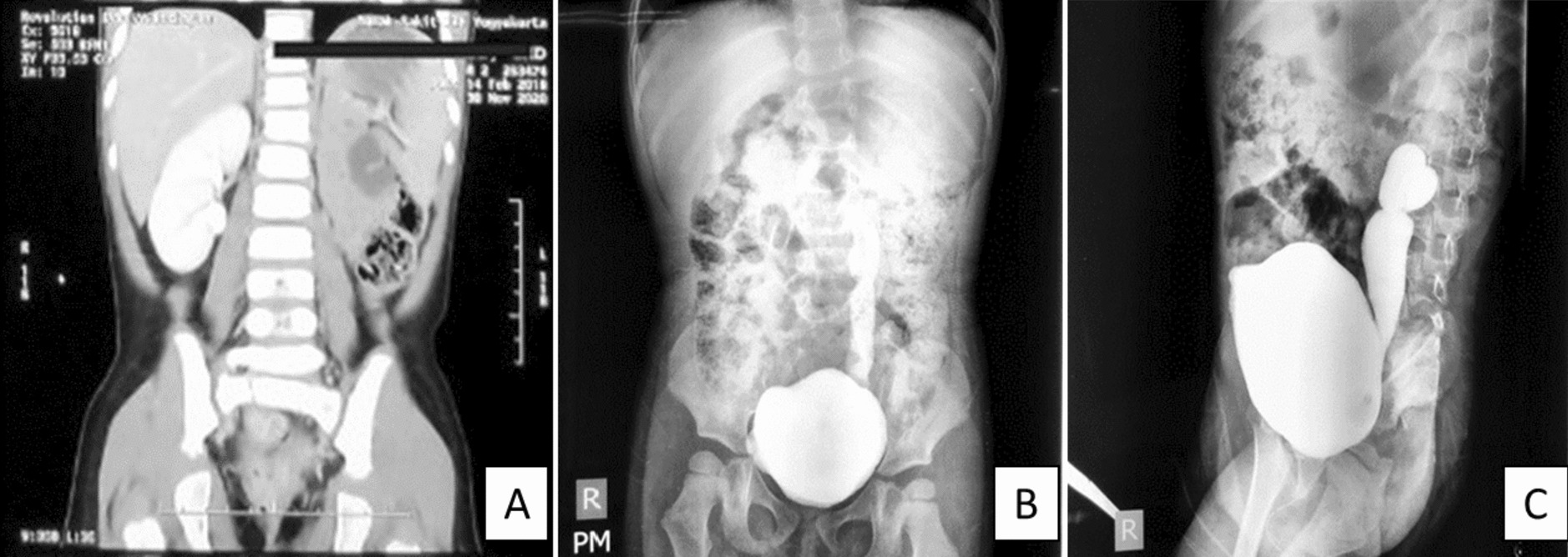
Radiological examination **A**. Abdominal CT-scan with contrast showed the absence of left kidney. **B**, **C** Voiding Cystourethrography revealed gross dilatation and ureteral tortuosity of left ureter (vesicoureteral reflux grade 5), without observed dilatation of right ureter (grade 1)

A 2 years old Javanese boy came with complaints of recurrent fever and urinary tract symptoms such as dysuria and straining. The patient was born via cesarean section at 34 weeks of gestation, with birth weight of 2250 g and considered as low birth weight. In his growth and development, this patient experienced numerous clinical problems in the form of abnormalities in his organs, such as atresia ani and unilateral undescended testicles. During the first year of his life, this patient underwent several definitive surgeries such as colostomy, anoplasty and orchidopexy. Right now this patient came with complaints of recurrent fever and urinary tract symptoms such as dysuria and straining. From a computerized tomography (CT) scan of the abdomen, the results showed there was an abnormality in the urinary tract (Fig. 1). Cystourethrography examination was done and showed grade 5 voiding and grade 1 vesicoureteral reflux on both the left and right side Fig. 1.

**Fig. 2:**
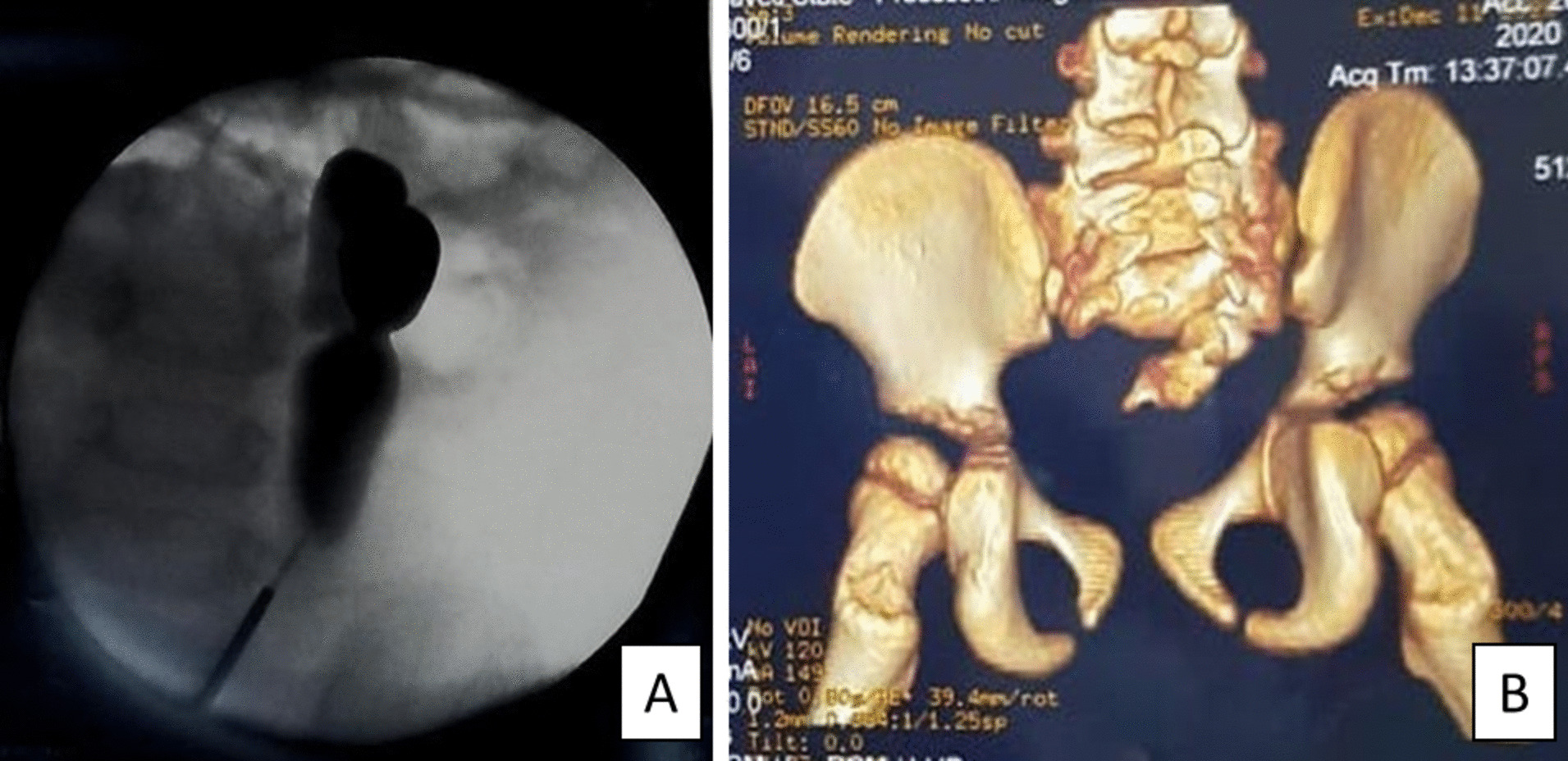
Radiological examination. **A** Retrograde Pyelography showed duplication of the left ureter and the absence of left collecting system. **B** MRI result showed bone defect at the posterior arch to confirm spina bifida

Retrograde pyelography is a necessary examination to confirm the diagnosis of unilateral renal agenesis. From this examination, it was found that there was a duplication in the left ureter and the absence of a collecting system on the left side. This patient also underwent a lumbosacral magnetic resonance imaging (MRI) examination to confirm whether this patient suffered from spina bifida or not (Fig. 2). A laparoscopic left ureterectomy examination was carried to see how the patient's urinary tract functions Fig. 2.

### Surgical procedure

**Fig. 3:**
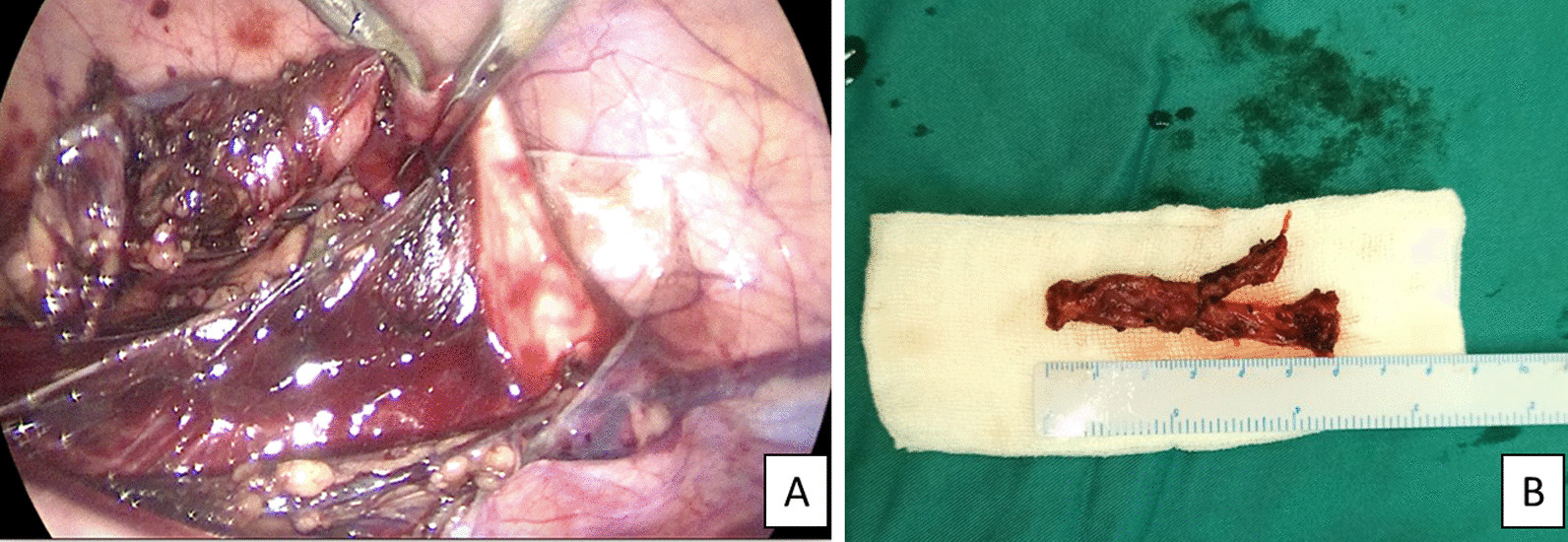
Surgical findings: **A** Transperitoneal laparoscopy of left ureterectomy. **B** Specimen of resected left ureter for histopathological analysis, showed no kidney tissue observed macroscopically

In the end, laparoscopic left transperitoneal ureterectomy was done because of the presence of recurrent infections and visible vesicoureteral reflux (VUR) grade 5. The procedure began with the insertion of three 3 mm ports in the left abdominal hemi. Followed by opening the white line to access the ureter. Blunt dissection and hemodynamic control were performed afterward. The left ureter was cut at a height to reach the vesicoureteral junction and macroscopically no kidney image remained at all in the resected tissue (Fig. 3). After that, the specimens were taken to the pathology laboratory for histopathological investigation Fig. 3.

### Histopathological finding

**Fig. 4:**
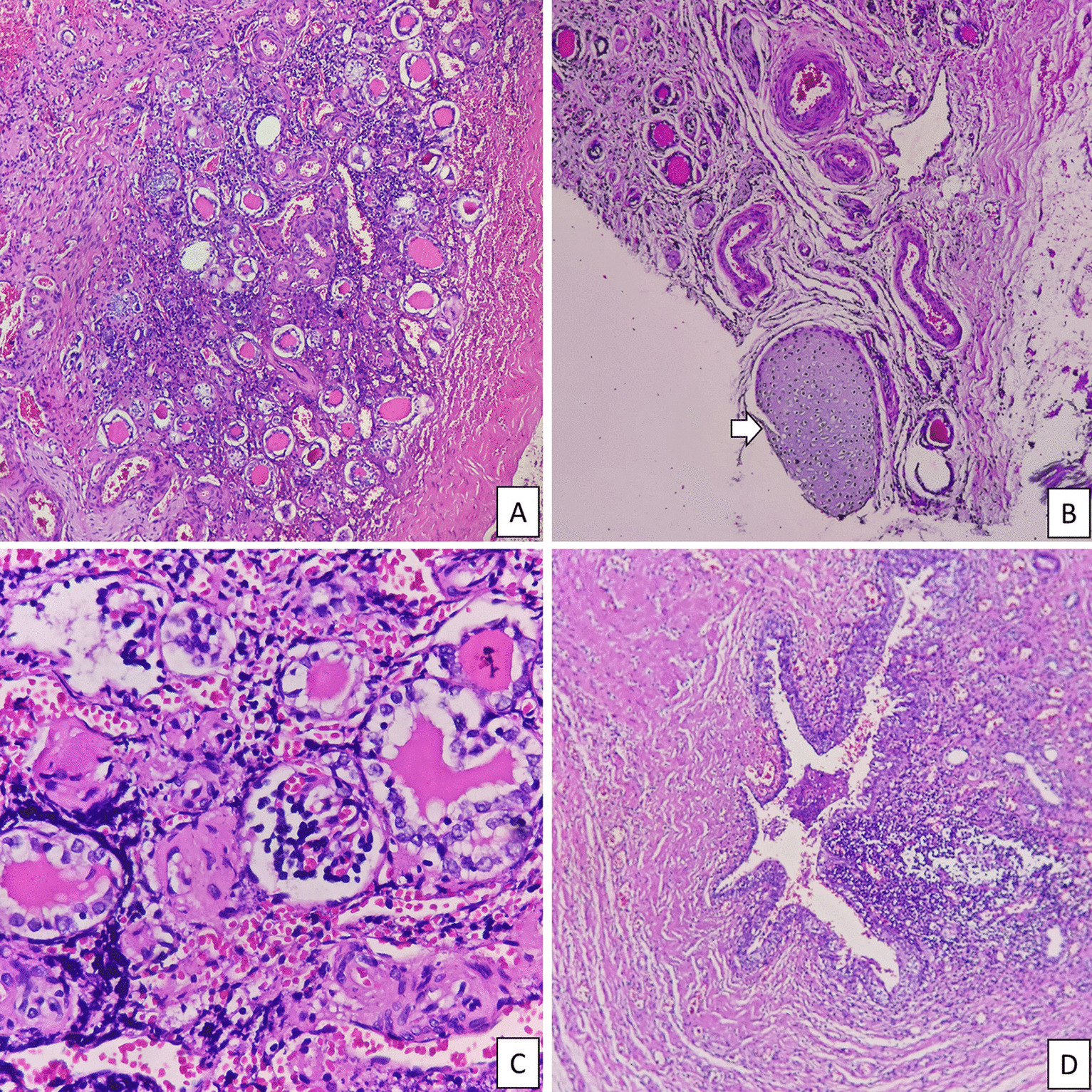
Histopathology examination. **A** The blunt-end ureter specimen microscopically showed primitive appearing tubules of renal tissue with dilated-atrophic tubule and colloid-like hyaline cast formation (thyroidization) (40x). **B** Fibrotic stroma with the chronic inflammatory cells and island of metaplastic cartilage were observed (white arrow) (100x). **C** The non-sclerotic glomeruli still can be observed (400x). **D** Urothelial cells lined proximal segment of ureter and infiltrated by chronic inflammatory cells (100x)

The results of the pathological examination concluded several things. Most of the primitive kidney tissue with parenchyma had been replaced with fibrotic tissue. In several places such as atrophic dilated kidney tubules and a small part of the glomerulus, colloid-like material (thyroidization) can be seen. This pathological examination does not identify the cortex and medulla of the kidney because it is quite difficult to process. However, foci of ureteral tissue with monomorphic urothelial cells were identified. Apart from that, the fibrous stroma is edematous and fibrotic with chronic inflammatory cells, mostly lymphocytes. The blastema component also cannot be seen, but metaplastic cartilage islands could be seen in our case Fig. 4.

### Immunohistochemical analysis

**Fig. 5:**
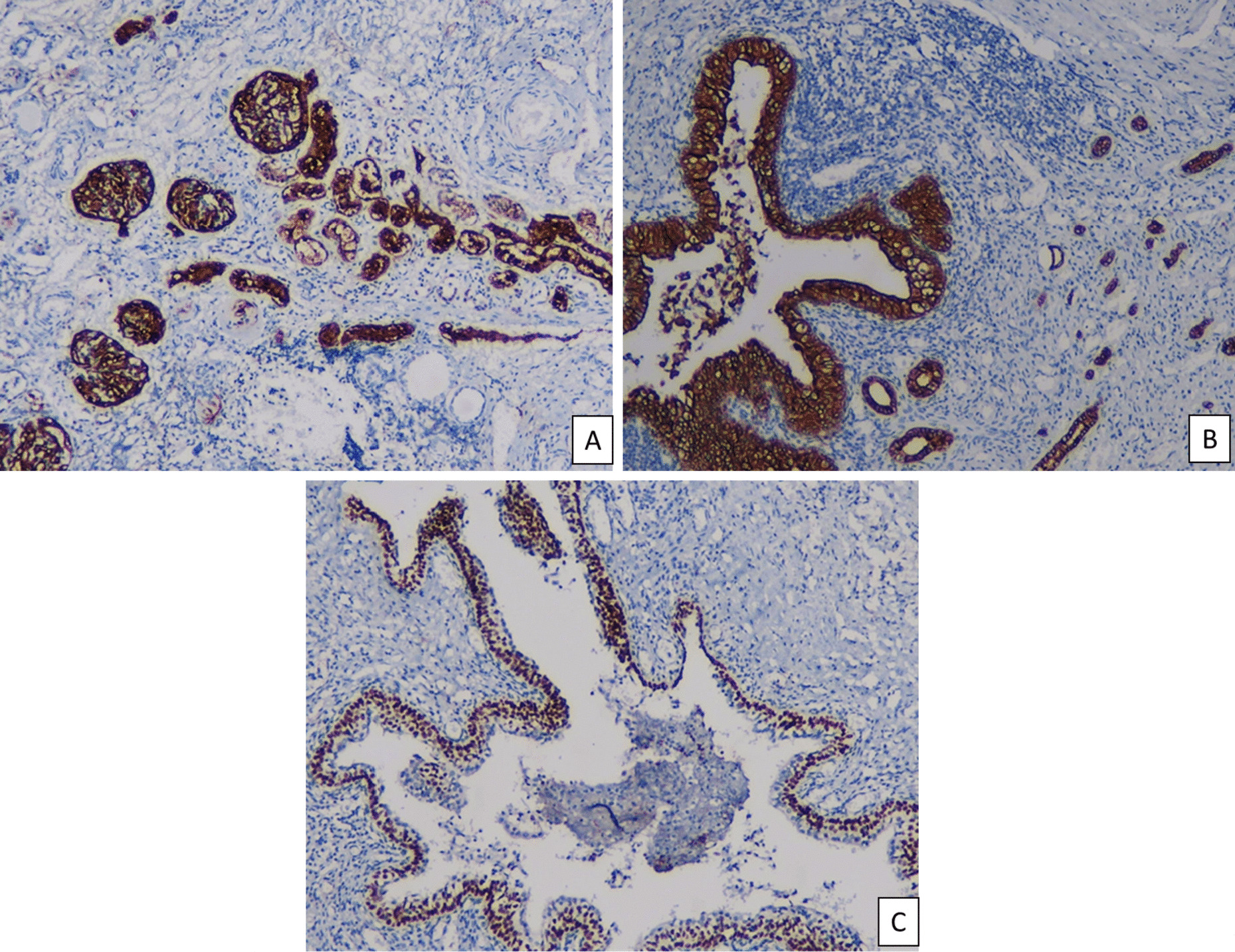
Immunohistochemical examination revealed strong expression of CD10 in glomeruli and tubules of renal tissue (**A**). Collecting duct and urothelial cells was strongly stained with CK7 (**B**), and basal cell of urothelium was positively expressed p63 (**C**)

Further examination immunostaining using CD10, CK7 and p63 which aims to check the presence of renal glomeruli and tubules, urothelial cells and urothelial basal cells. The examination results of each immunostaining test included, the CD10 test gave positive staining results in the glomerulus and remaining renal tubular cells, as well as very positive CK7 staining in urothelial cells and positive p63 expression in urothelial basal cells. The conclusion that can be drawn is that after the initial examination was carried out and the diagnosis was established in the form of unilateral renal agenesis, the diagnosis changed because the results of histopathological and immunohistochemical examinations were more likely to lead to a diagnosis of severe dysplasia or aplastic kidney Fig. 5.

### Post-surgical condition

After the surgical management was carried out, the condition of the patient showed positive results because there was a complete resolution of the condition of fever and dysuria which had been the main complaint so far. Video urodynamic studies were performed two months after surgery. The results show that the patient's bladder capacity is 120 cc. From the examination, it was concluded that this patient was already experiencing a normal condition because there were no abnormalities found when the filling phase had reached 120 cc, grade I right ureteral reflux, detrussor overactivity, normal bladder compliance and bladder capacity. When it was time to urinate, the patient was able to void 75 cc of urine and 75 cc of post-voiding volume. For now, the condition of spina bifida experienced by patients is still the responsibility of the neurosurgeon for follow-up and management.

## Discussion

The mechanism of renal dysplasia is explained in two principles: (1) In 1964, Osathondh and Potter theorized that renal dysplasia could result from abnormal activity of the ampullary which leads to insufficient induction of nephrons in both types 2 and 4. (2) The second is the “Budding Theory” by Stephens et al., theorizing that if the ureteral bud from an ectopic site with a metanephric blastema can cause bad contact, it will result in metanephric development which is far from normal [6].

Kidney dysplasia that occurs in extreme cases can occur in the form of kidney involution known as rudiments. This phenomenon is the result of an aplastic kidney which may not be detected radiologically, which leads to a diagnosis of solitary kidney or unilateral agenesis. There is a study that has been reported on how renal aplasia is a cause of congenital solitary kidney [5]. In this case, renal aplasia occurred in a boy who was born with a congenital condition of imperforate anus and unilateral undescended testes. In previous studies, it was reported that the incidence of cryptochismus goes hand in hand with the incidence of anorectal malformations. As well as the occurrence of renal or ureteral malformations and dysplasia shows a trend in parallel [7].

The definition of renal dysplasia begins with a histopathological examination where abnormal tissue is found in the kidney. The American Urological Association reports three classic features of renal dysplasia: “cuffs” or spindle cells around primitive or fetal-looking tubules, islands of metaplastic cartilage (30% of cases), and loss of normal renal architecture [8]. From a macroscopic point of view, kidney size can be said to be quite variable [9].

Aplastic or dysplastic kidney conditions can be differentially diagnosed from conditions such as kidney agenesis, atrophy, or hypoplasia. The importance of histopathological examination lies in establishing and confirming a valid diagnosis, differentiating between various possible etiologies of kidney dysplasia.. This aims to determine how the prognosis and genetic testing need to be done. There are several markers that can help histopathological examination so that the diagnosis is upright and valid, such as PAX2/8, WT1, CD10, CK7 and P63. The action of strong expression of PAX2/8 markers in primitive ducts and WT1 in fibromuscular [4]. The CD10 marker can help express all stages of human kidney development because since the stages of embryogenesis, the kidney is able to help the examination to detect the presence of tubular and glomerular epithelium [10]. The CK7 marker also helps establish the diagnosis because it can detect the renal collecting ducts and urothelial cells. Then there is the marker p63 which is strongly expressed in the basal cells of the urothelium [11, 12].

It is safe to say that the condition of unilateral kidney dysplasia or aplasia will have a good prognosis if the contralateral kidney is in good condition [13]. In the disease development process of Chronic Renal Failure (CRF) in children suffering from dysplastic or aplastic conditions, is divided into three periods: (a) In the first three years of life, children tend to have stable and good kidney function; (b) For an average of 8 years the patient has a stable condition of renal function covering 50% of the patient population; and (c) The final phase in which kidney function may deteriorate to the stage of end-stage renal disease.

Postnatal glomerular filtration rate (GFR) has been shown to have predictive value. GFR < 15 mL/min per 1.73 m^2^ at 6 months or < 25 mL/min per 1.73 m^2^ at 18 months were more likely associated with a poorer prognosis [14]. In this case, the reason for not performing the GFR test was because the serum creatinine level and diuresis results were in the normal stage.

In many cases of kidney dysplasia or kidney aplasia, the preferred treatment is nephrectomy or ureteral resection. But a retrospective cohort study says the opposite. Due to a decrease in the percentage of surgery performed on dysplastic kidney disease in children’s hospitals, which was initially in 2006 at 22.1% and in 2015 it became 7.3% [15]. In recent times, conservative management with careful follow-up has been considered. Mainly shown in limited conditions in one kidney only and no symptoms. One of the follow-up actions taken is to carry out an ultrasound examination in order to examine the kidneys, both the affected kidney and the contralateral kidney. Consideration of surgical management still needs to be considered, especially if the patient’s quality of life is compromised due to significant symptoms. In our case, the reason we treated the patient with the surgical stage of left ureteral resection was because of symptoms that affected the patient's life, such as recurrent fever, dysuria and straining.

The use of Angiotensin-converting enzyme (ACE) inhibitors has a function to improve kidney function [4]. The use of ACE inhibitors can be used in patients with CRF due to dysplasia or aplasia of the kidney, with or without reflux. Using ACE inhibitors can increase GFR up to 40–50 mL/minute. In our patient, because the patient did not occur or showed symptoms that led to the occurrence of CRF, we did not prescribe ACE inhibitors.

In handling and management processes in cases of aplastic kidney will be adjusted to the age of the patient. If the patient is an adult, the treatment process that will be recommended includes limiting salt consumption, asking the patient to reduce excessive protein intake, regular and periodic evaluation of blood pressure, uric albumin and serum creatinine. It is different in children who have conditions of congenital abnormalities of the kidneys and urinary tract. Several parameters must be monitored and evaluated twice a year. Ultrasound examinations that are carried out routinely have the aim that this compensated hypertrophy condition is in accordance with the patient’s growth into adolescence [16]. In our patient, urinalysis and serum creatinine examinations had to be evaluated every 3 months, as well as checking urinary function with VCUG (Voiding Cystourethrography) which was only done 3 months postoperatively.

## Conclusions

From this case report, it was observed that cases of unilateral renal aplasia mimic those of renal agenesis radiologically and macroscopically. This observation precisely stems from histopathological and immunohistochemical examinations directing the diagnosis towards kidney aplasia due to the discovery of primitive kidney structures. An accurate diagnosis is necessary because managing the patient in accordance with the diagnosis will enhance the patient’s quality of life.

## Data Availability

Data sharing was not applicable to this article, as no datasets were generated or analysed during the current study.

## Abbreviations

URA: Unilateral renal agenesis
VATER: Vertebral, anorectal, tracheoesophageal, and renal
MURCS: Mullerian duct aplasia, uterine hypoplasia, renal aplasia, and cervicothoracic somite dysplasia
VUR: Vesicoureteral reflux
CRF: Chronic renal failure
